# circRNA-UBAP2 promotes the proliferation and inhibits apoptosis of ovarian cancer though miR-382-5p/PRPF8 axis

**DOI:** 10.1186/s13048-020-00685-w

**Published:** 2020-07-20

**Authors:** Qin Xu, Bo Deng, Manlin Li, Yang Chen, Li Zhuan

**Affiliations:** 1 Department of Reproductive Medicine, Reproductive Medical Centre, The First People’s Hospital of Yunnan Province, No. 157 Jin Bi Road, Kunming, People’s Republic of China; 2 Kunming University of Science and Technology, Kunming, People’s Republic of China

**Keywords:** circRNA-UBAP2, miR-382-5p, PRPF8, Proliferation, Apoptosis, Ovarian cancer

## Abstract

**Objective:**

circular RNAs (circRNAs) have been reported to be essential regulators of multiple malignant cancers. However, the functions of circRNAs in ovarian cancer need to be further explored. The aim of our study is to explore the role of circRNA-UBAP2 in ovarian cancer and its mechanism.

**Results:**

circRNA-UBAP2 was upregulated in ovarian cancer tissues and cell lines. Knockdown of circRNA-UBAP2 inhibited cell proliferation and promoted cell apoptosis, but circRNA-UBAP2 overexpressed got opposite results. In addition, circRNA-UBAP2 targeted miR-382-5p and downregulated its expression, PRPF8 was a target gene of miR-382-5p. Furthemore, circRNA-UBAP2/miR-382-5p/PRPF8 axis affected the proliferation, apoptosis and cell cycle of ovarian cancer through the mechanism of competing endogenous RNAs (ceRNA).

**Conclusion:**

circRNA-UBAP2 acted as a ceRNA to sponged miR-382-5p, increased the expression level of PRPF8, and prompted proliferation and inhibited apoptosis in ovarian cancer cells.

## Background

Ovarian cancer is one of the most common gynecologic malignancy in the world [1]. However, it generally presents at an advanced stage which is one of the main factors contributing to the high death to incidence rate [2]. Although operation, combined chemotherapy and radiotherapy play an important role in the treatment of ovarian cancer, 75% patients have experienced a cancer recurrence, and malignant proliferation is one of the major poor prognostic factors for ovarian cancer [3]. Inhibiting ovarian cancer cell malignant proliferation is very important for the treatment, thus it becomes particularly urgent to find the gene target which regulates the proliferation and apoptosis of ovarian cancer cells.

Recently many studies conformed that circRNAs play an important role in the malignant biological behavior of cancer cells. For example, circRNA-UBAP2 effects the proliferation and invasion of lung cancer cells [4], circRNA-UBAP2 also serves as a promising therapeutic target for triple-negative breast cancer patients [5]. But the role of circRNA-UBAP2 in ovarian cancer remains to be further explored. Besides, even a great deal of researches on the genesis and development of ovarian cancer have been done, but the pathophysiology of ovarian cancer development is complex and involves numerous biological pathways [6], the etiology of ovarian cancer is poorly understood. This study explored how circRNA-UBAP2 affects the ovarian cancer and its mechanisms, we hoped that our study will support the use of circRNA-UBAP2 as prognostic and predictive markers in ovarian cancer.

circRNAs may use their circularization to competitively inhibit the linear splicing and sponge function of microRNAs (miRNAs), and modulate the expression of metastasis-evoking genes, thus contributing to the development of cancer. It was reported that miR-382-5p may engaged in various cancers. For instance, there was an upregulation of miR-382-5p in primary myelofibrosis cells [7], but the expression of miR-382-5p was downregulated in glioma-exposed endothelial cells [8]. But there have been no studies about circRNA-UBAP2 regulated miR-382-5p.

miRNAs as a small non-coding RNAs can participate in the cancer process through modulate post-transcriptional gene expression. Whether miRNA is an oncogene or anti-oncogene was determined by its target mRNA. Disruption of RNA splicing causes genome instability, which could contribute to cancer etiology [9]. As RNA splicing becomes an emerging anti-cancer target, pre-mRNA processing factor 8 (PRPF8) which is the core protein of splicing has been extensively studied in recent years. Knockdown of pan-cancer drivers PRPF8 caused cell apoptosis in breast cancer, which dedicated that PRPF8 is a proto-oncogene regulating cell viability [10]. However, there is no report about the miR-382-5p targeted PRPF8 and effect on ovarian cancer.

In this study, we found that circRNA-UBAP2 was highly expressed in ovarian cancer tissues and cell lines. Then we assessed that circRNA-UBAP2 promoted the malignant biological behavior of ovarian cancer cells in vitro. The mechanism that circRNA-UBAP2 promoted cell proliferation and inhibited cell apoptosis in ovarian cancer cells was regulated miR-382-5p/PRPF8 axis. This article was written to provide a molecular target for early diagnosis and treatment of ovarian cancer.

## Results

### circRNA-UBAP2 was upregulated in ovarian cancer tissues and cell lines

**Fig. 1:**
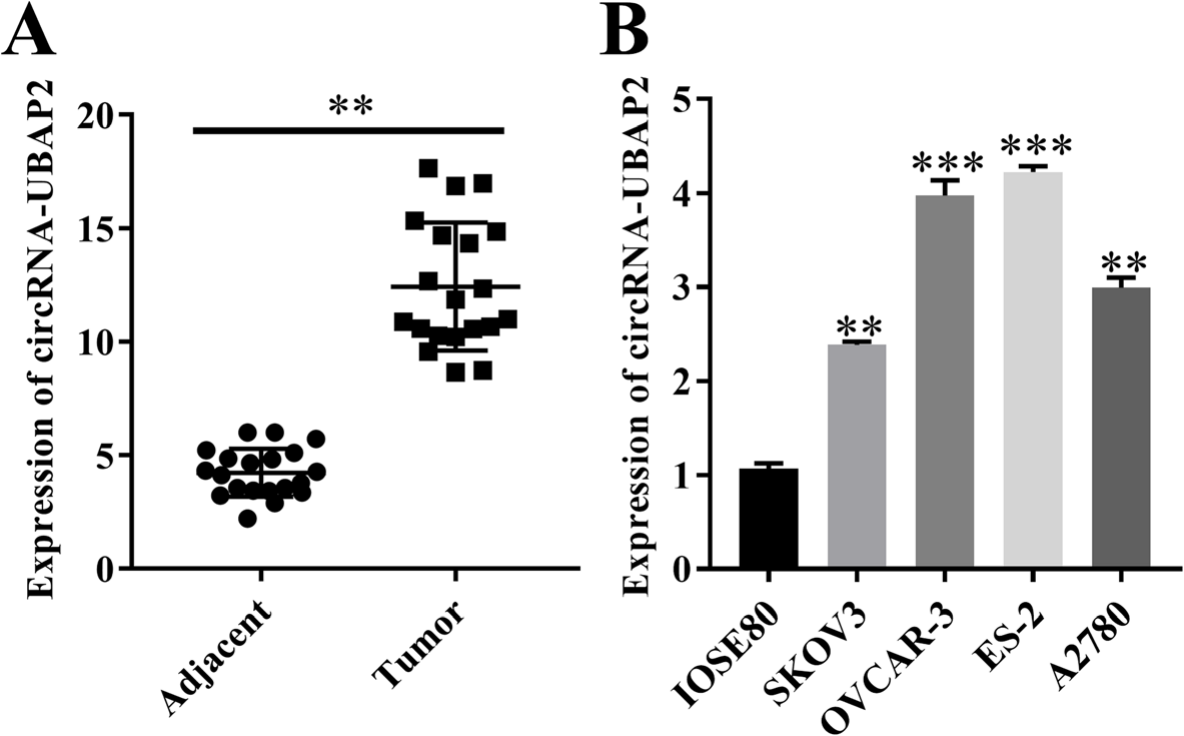
circRNA-UBAP2 was upregulated in ovarian cancer tissues and cell lines. **a**, Differentially expressed circRNA-UBAP2 in ovarian cancer tissues and adjacent tissues were measured by RT-qPCR assay. ^**^P < 0.01 vs adjacent tissues. **b**, Relative expression of circRNA-UBAP2 in normal ovarian epithelial cells (IOSE80) and ovarian cancer cells (SKOV3, OVCAR-3, ES-2 and A2780) were measured by RT-qPCR assay. ^**^P < 0.01, ^***^P < 0.001 vs normal epithelial cells (IOSE80) group

circRNAs act as an oncogene in a variety of tumors, we measured the expression level of circRNA-UBAP2 in ovarian cancer tissues and cell lines by RT-qPCR assay, the results showed that expression level of circRNA-UBAP2 was significant higher in ovarian cancer tissues than that in adjacent tissues (Fig. 1a). Moreover, the expression levels of circRNA-UBAP2 was markedly upregulated in ovarian cancer cells compared with normal ovarian epithelial cells, especially in OVCAR-3 and ES-2 (Fig. 1b). Thus, the above results showed that circRNA-UBAP2 was highly expressed in ovarian cancer tissues and cell lines, and we chose OVCAR-3 and ES-2 to do further experiments.

### circRNA-UBAP2 regulated the malignant biological behavior of ovarian cancer cells

**Fig. 2:**
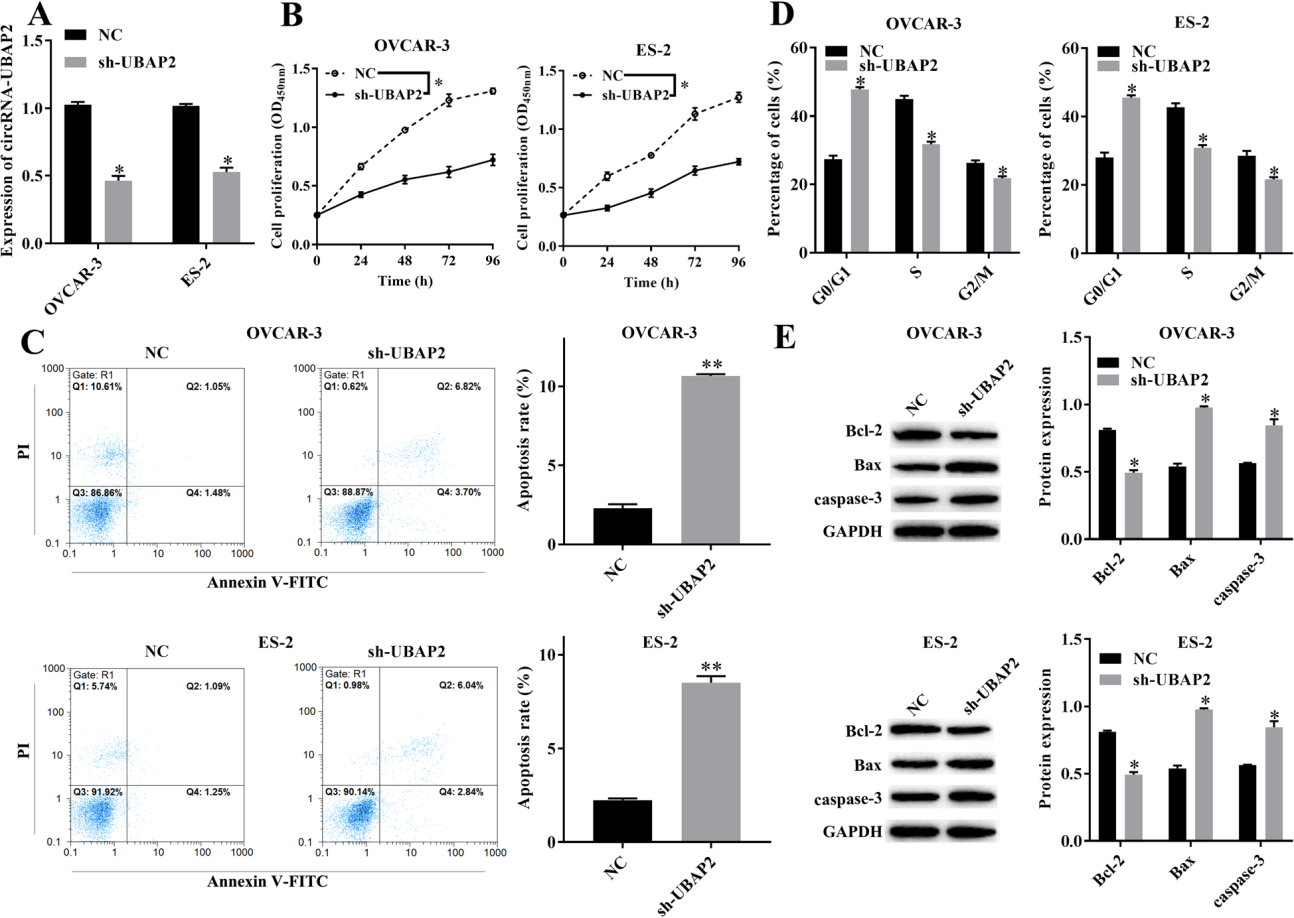
Knockdown of circRNA-UBAP2 (sh-UBAP2) suppressed the malignant biological behavior of ovarian cancer cells. **a**, Relative expression of circRNA-UBAP2 in OVCAR-3 and ES-2 were reduced after transfected sh-UBAP2 measured by RT-qPCR assay. ^*^P < 0.05 vs NC group. **b**, Proliferation of OVCAR-3 and ES-2 cells were inhibited after transfected sh-UBAP2 measured by CCK-8 assay. ^*^P < 0.05 vs NC group. **c**, Apoptosis of OVCAR-3 and ES-2 cells were promoted after transfected sh-UBAP2 measured by flow cytometry. ^*^P < 0.05, ^**^P < 0.01 vs NC group. **d**, Cell cycle of OVCAR-3 and ES-2 cells were influenced after transfected sh-UBAP2 measured by flow cytometry. ^*^P < 0.05 vs NC group. **e**, Apoptosis-related proteins Bcl-2, Bax and caspase-1 of OVCAR-3 and ES-2 cells were upregulated after transfected sh-UBAP2 measured by western blotting. ^*^P < 0.05 vs NC group

**Fig. 3:**
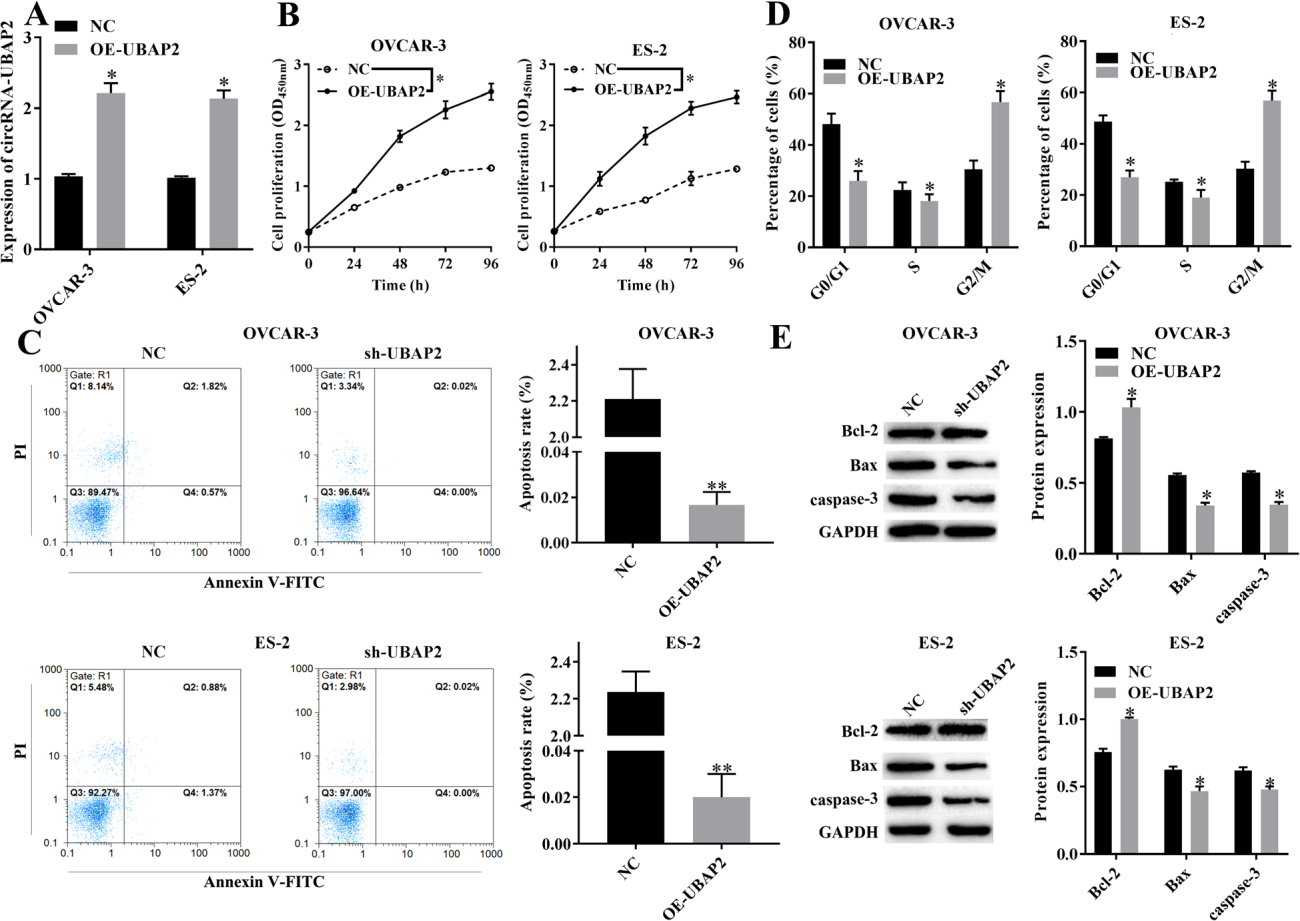
Overexpression of circRNA-UBAP2 (OE-UBAP2) promoted proliferation and inhibited apoptosis of ovarian cancer cells. **a**, Relative expression of circRNA-UBAP2 in OVCAR-3 and ES-2 were upregulated after overexpressed UBAP2 measured by RT-qPCR assay. ^*^P < 0.05 vs NC group. **b**, Proliferation of OVCAR-3 and ES-2 cells were remarkably promoted after overexpressed UBAP2 measured by CCK-8 assay. ^*^P < 0.05 vs NC group. **c**, Apoptosis of OVCAR-3 and ES-2 cells were inhibited after overexpressed UBAP2 measured by flow cytometry. ^*^P < 0.05, ^**^P < 0.01 vs NC group. **d**, Cell cycle of OVCAR-3 and ES-2 cells were affected after overexpressed UBAP2 measured by flow cytometry. ^*^P < 0.05 vs NC group. **e**, The expression of Apoptosis-related proteins Bcl-2, Bax and caspase-1 of OVCAR-3 and ES-2 cells were decreased after overexpressed UBAP2 measured by western blotting. ^*^P < 0.05 vs NC group

Based on the high expression of circRNA-UBAP2 in ovarian cancer tissues and cell lines, in order to further explored the effect of circRNA-UBAP2 on biological behavior of OVCAR-3 and ES-2 cells, we either knockdown or overexpression circRNA-UBAP2 in OVCAR-3 and ES-2 cells, and verified the expression level of circRNA-UBAP2 by RT-qPCR in both OVCAR-3 and ES-2 cells (Figs. 2a, 3a). CCK-8 showed that knockdown of circRNA-UBAP2 inhibited the proliferation of OVCAR-3 and ES-2 cells (Fig. 2b). Moreover, flow cytometry results showed that knockdown of circRNA-UBAP2 promoted cell apoptosis of OVCAR-3 and ES-2 (Fig. 2c), as well as arrested cell cycle at G0/G1 phase (Fig. 2d). Besides, western blotting showed that knockdown of circRNA-UBAP2 decreased the expression level of anti-apoptotic protein Bcl-2, while increased the pro-apoptotic proteins Bax and caspase-3 in OVCAR-3 and ES-2 (Fig. 2e). Importantly overexpression of circRNA-UBAP2 had the opposite effect (Fig. 3b-e). Therefore, knockdown of circRNA-UBAP2 significantly inhibited cell proliferation and promoted cell apoptosis in these two ovarian cancer cells.

### circRNA-UBAP2 targeted miR-382-5p

**Fig. 4:**
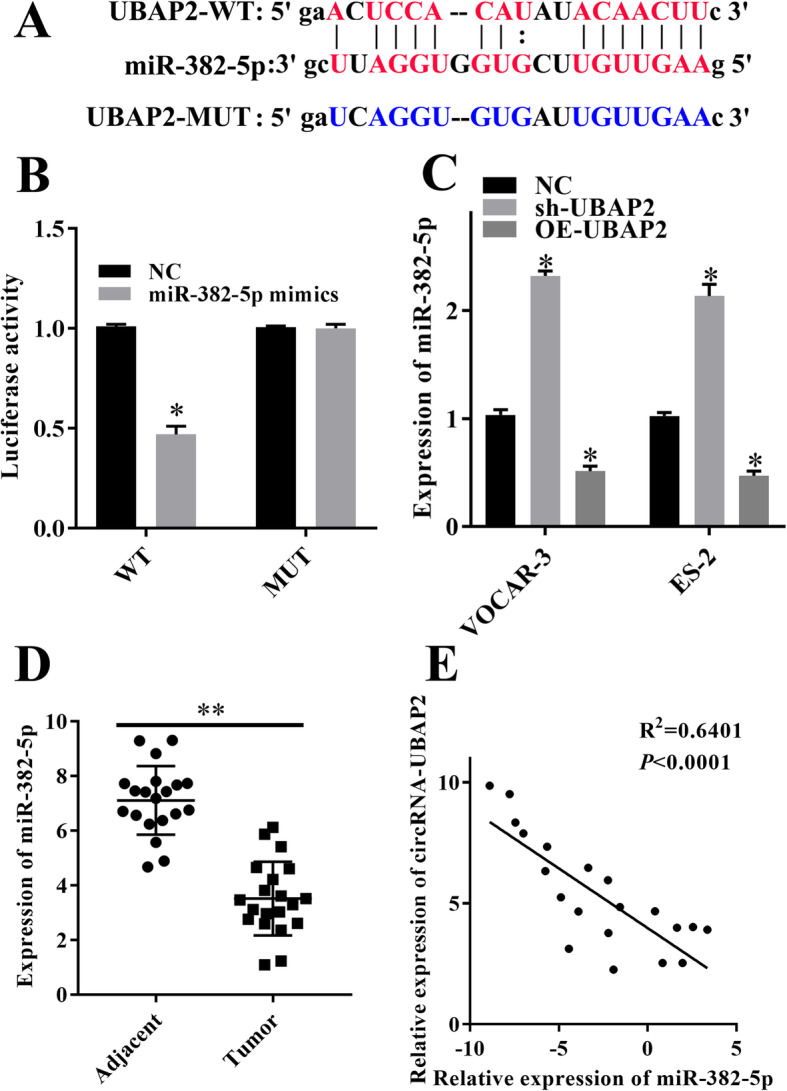
circRNA-UBAP2 targeted miR-382-5p. **a**, The binding site of miR-382-5p and circRNA-UBAP2 was predicted by starBase. **b**, The interaction of circRNA-UBAP2 and miR-382-5p was determined by dual-luciferase reporter gene system. ^*^P < 0.05 vs NC group. **c**, Knockdown and overexpression of circRNA-UBAP2 effected on the expression of miR-382-5p in SKOV3 and A2780 cells measured by RT-qPCR assay. ^*^P < 0.05 vs NC group. **d**, Expression of miR-382-5p in ovarian cancer tissues and adjacent tissues were measured by RT-qPCR assay. ^**^P < 0.01 vs adjacent tissues. **e**, The expression level of circRNA-UBAP2 was negatively related to miR-382-5p in ovarian cancer tissues measured by RT-qPCR assay. *P* < 0.0001

Prediction results of bioinformatics database showed that miR-382-5p was one of the target genes of circRNA-UBAP2 (Fig. 4a). Dual-luciferase reporter gene system results showed that overexpression of miR-382-5p decreased the luciferase activity of circRNA-UBAP2 WT reporter, but no effect on the circRNA-UBAP2 MUT reporter (Fig. 4b). Besides, RT-qPCR showed that downregulated of circRNA-UBAP2 remarkably increased the expression levels of miR-382-5p in both OVCAR-3 and ES-2 cells, and overexpression of circRNA-UBAP2 significantly decreased the expression of miR-382-5p (Fig. 4c). Moreover, compared with the adjacent tissues, the expression level of miR-382-5p was significant lower in ovarian cancer tissues (Fig. 4d). Thus, we confirmed that circRNA-UBAP2 sponged miR-382-5p and reduced the expression of miR-382-5p in both ovarian cancer tissues and cell lines.

### miR-382-5p targeted PRPF8

**Fig. 5:**
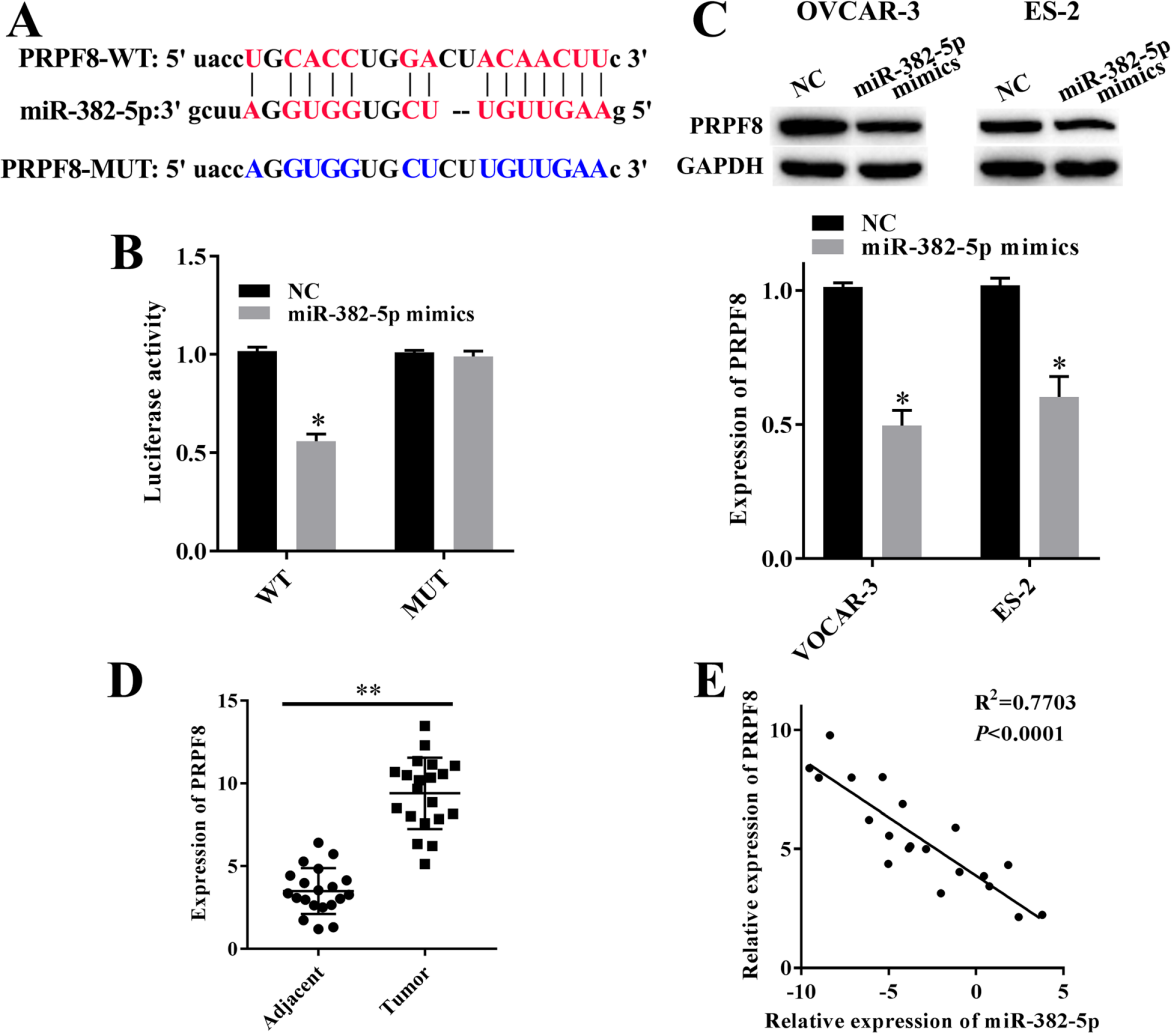
miR-382-5p targeted PRPF8. **a**, The binding site of miR-382-5p and PRPF8 was predicted by starBase. **b**, The interaction of PRPF8 and miR-382-5p was determined by dual-luciferase reporter gene system. ^*^P < 0.05 vs NC group. **c**, Overexpression of miR-382-5p decreased the expression of PRPF8 in SKOV3and A2780 cells measured by western blotting. ^*^P < 0.05 vs NC group. **d**, Expression of PRPF8 in ovarian cancer tissues and adjacent tissues were measured by RT-qPCR assay. ^**^P < 0.01 vs adjacent tissues. **e**, There was a negative correlation existed between expression level of PRPF8 and miR-382-5p in ovarian cancer tissues measured by RT-qPCR assay. *P* < 0.0001

Further we confirmed that miR-382-5p targeted PRPF8 (Fig. 5a). And overexpressed miR-382-5p decreased the luciferase activity of PRPF8 WT reporter, but not the PRPF8 MUT reporter (Fig. 5b). Besides, western blotting showed that overexpression of miR-382-5p reduced the expression levels of PRPF8 in OVCAR-3 and ES-2 cells (Fig. 5c). Additionally, RT-qPCR result showed that the expression of PRPF8 was higher in ovarian cancer tissues than that in adjacent tissues (Fig. 4d). Moreover, there was a negative correlation existed between expression level of PRPF8 and miR-382-5p in ovarian cancer tissues (Fig. 4e). Thus, PRPF8 was one of the target genes of miR-382-5p in OVCAR-3 and ES-2.

### circRNA-UBAP2 regulated cell proliferation and apoptosis of ovarian cancer through miR-382-5p/PRPF8 axis

**Fig. 6:**
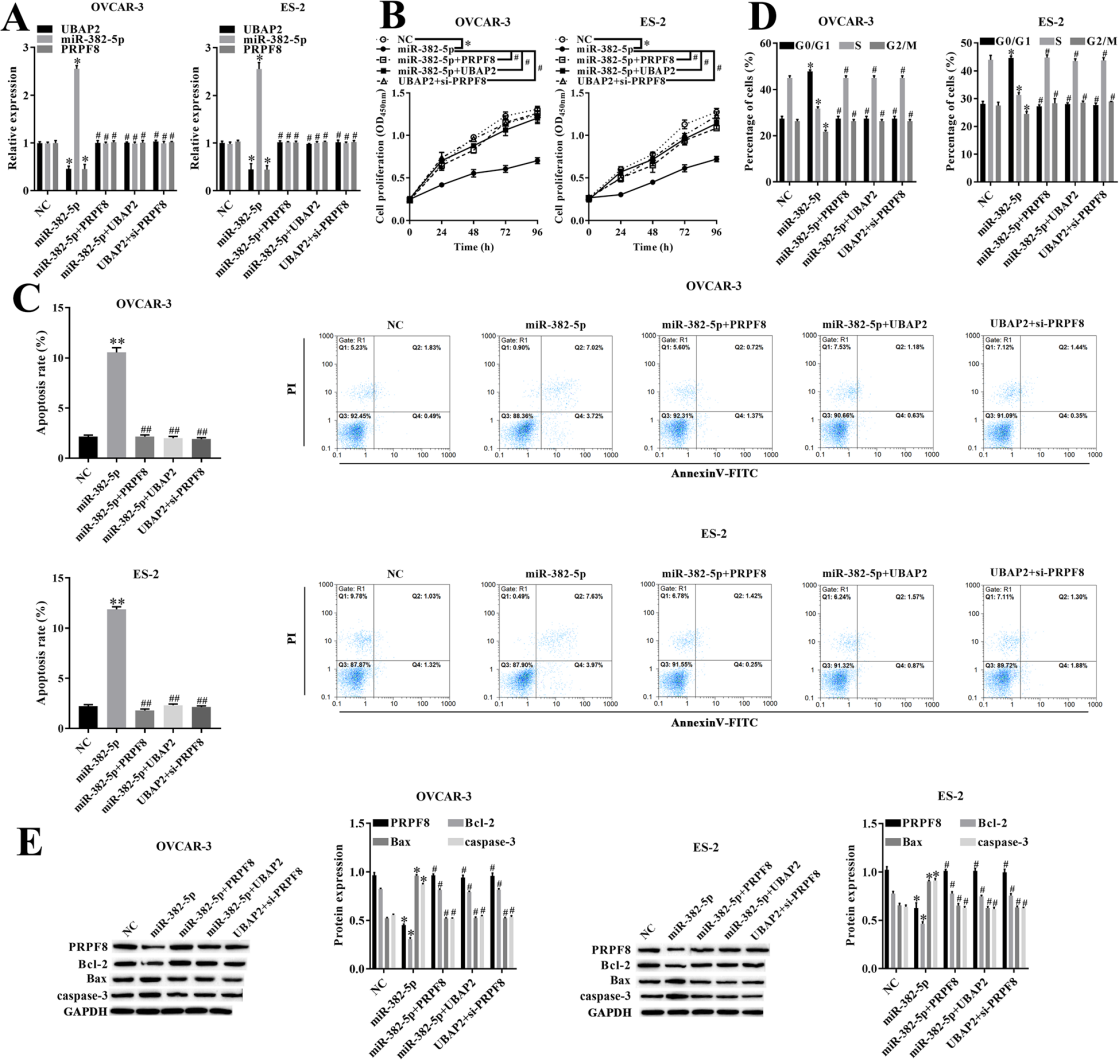
circRNA-UBAP2 regulated cell proliferation and apoptosis of ovarian cancer through miR-382-5p/PRPF8 axis. **a**, Relative expression of UBAP2, miR-382-5p and PRPF8 in OVCAR-3 and ES-2 were measured by RT-qPCR assay. ^*^P < 0.05 vs NC group. **b**, Proliferation of OVCAR-3 and ES-2 cells were measured by CCK-8 assay. ^*^P < 0.05 vs NC group. ^*#*^*P* < 0.05 vs miR-382-5p group. **c**, Apoptosis of OVCAR-3 and ES-2 cells were measured by flow cytometry. ^*^P < 0.05, ^**^P < 0.01 vs NC group. ^*#*^*P* < 0.05, ^*##*^*P* < 0.05 vs miR-382-5p group. **d**, Cell cycle of OVCAR-3 and ES-2 cells were measured by flow cytometry. ^*^P < 0.05 vs NC group. ^*#*^*P* < 0.05 vs miR-382-5p group. **e**, Apoptosis-related proteins Bcl-2, Bax and caspase-1 of OVCAR-3 and ES-2 cells were measured by western blotting. ^*^P < 0.05 vs NC group. ^*#*^*P* < 0.05 vs miR-382-5p group

To further verified the mechanism that circRNA-UBAP2 regulated cell proliferation and apoptosis of ovarian cancer through miR-382-5p/PRPF8 axis, the relative expression of UBAP2, miR-382-5p and PRPF8 in each groups were measured by RT-qPCR at first (Fig. 6a). CCK-8 showed that overexpression of miR-382-5p significantly inhibited proliferation in OVCAR-3 and ES-2 cells compared with NC group. However, overexpressed miR-382-5p and PRPF8, overexpressed miR-382-5p and circRNA-UBAP2 or overexpressed circRNA-UBAP2 but downregulated PRPF8 at the same time alleviated the inhibition in cell proliferation by only overexpressed miR-382-5p (Fig. 6b). What was more, flow cytometry results showed that overexpressed miR-382-5p promoted cell apoptosis (Fig. 6c) and arrested cell in G0/G1 phase (Fig. 6d), but the results of the other three experimental groups showed no significant difference from the NC group. Additionally, western blotting showed that overexpressed miR-382-5p decreased the expression level of PRPF8 and Bcl-2, while overexpressed miR-382-5p increased the expression level of Bax and caspase-3 (Fig. 6e), nevertheless, the other three experimental groups showed opposite results. Consequently, circRNA-UBAP2 upregulated expression level of PRPF8 by sponged miR-382-5p acted as a ceRNA, promoted proliferation and inhibited apoptosis in ovarian cancer cells.

## Discussion

**Fig. 7:**
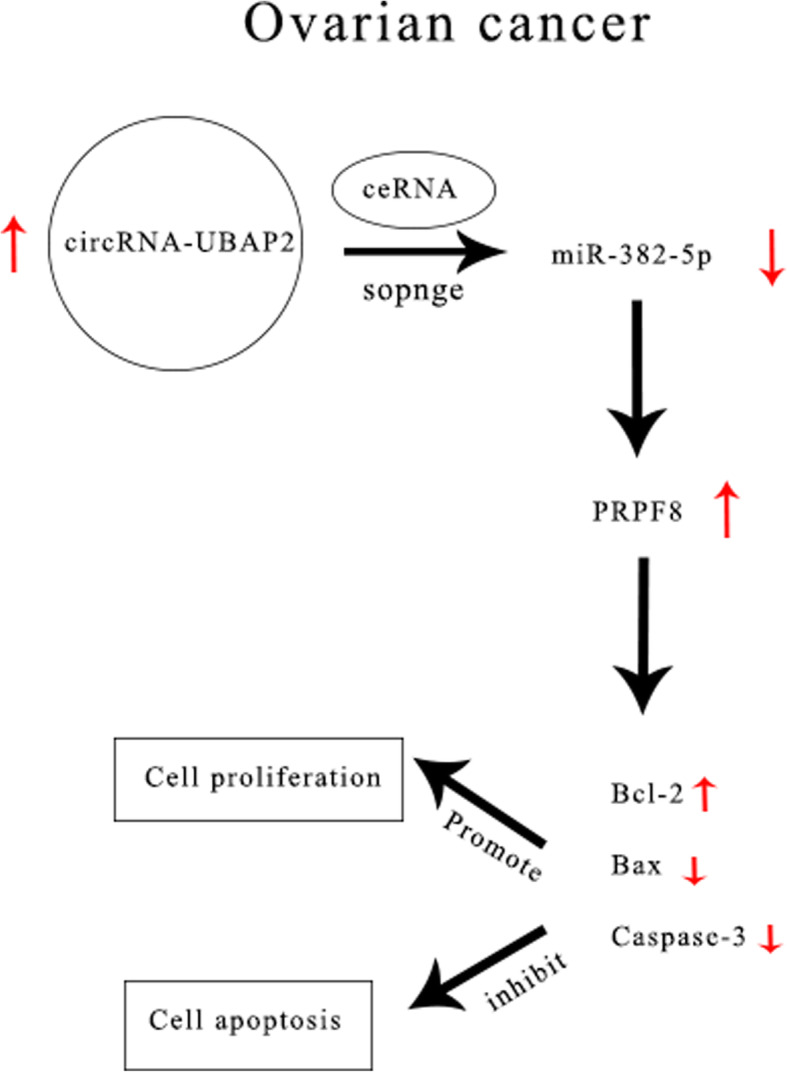
Schematic diagram of mechanism of this research. circRNA-UBAP2 was highly expressed in ovarian cancer, and UBAP2 upregulated PRPF8 by sponged miR-382-5p acted as a ceRNA, which led to Bcl-2 increased, Bax and Caspase-3 decreased, then promoted proliferation and inhibited apoptosis in ovarian cancer cells

Ovarian cancer is one of the most malignant tumours and a major cause of the high cancer-related mortality rate in women, thus it is important to find a better therapeutic method for ovarian cancer. In the present study, we conformed that circRNA-UBAP2 was upregulated in ovarian cancer tissues and cell lines, and circRNA-UBAP2 promoted cell proliferation and inhibited cell apoptosis of ovarian cancer through miR-382-5p/PRPF8 axis (Fig. 7).

More and more researches conformed that circRNAs play key regulatory roles in cancer progression [11]. Such as the overall survival time of non-small cell lung cancer patients with high circRNA_100876 expression was significantly shorter than those patients with low circRNA_100876 expression [12]. And circRNA_102,958 was significantly overexpressed in gastric cancer and it may be a potential novel and stable biomarker for the diagnosis of gastric cancer [13]. Besides, circRNA-RAPGEF5 contributes to papillary thyroid cancer cell proliferation, invasion, and migration [14]. Moreover, overexpressed circular RNA-MTO1 suppresses breast cancer cell viability and reverses monastrol resistan [15]. Studies also found that circRNAs regulated the malignant biological behavior of ovarian cancer cells [16]. For example, circEXOC6B and circN4BP2L2 may act as novel prognostic biomarkers in patients with epithelial ovarian cancer [17], circ_0051240 promotes cell proliferation, migration and invasion in ovarian cancer [18], and circ-HIPK3 is an important regulator of ovarian cancer progression [19]. Besides, our study found that circRNA-UBAP2 was highly expressed in ovarian cancer tissues and cell lines. Knockdown of circRNA-UBAP2 suppressed the malignant behavior of ovarian cancer cells, while overexpression of circRNA-UBAP2 promoted proliferation and inhibited apoptosis in ovarian cancer cells.

One of the methods that circRNAs affect the malignant biological behavior of cancer cell was circRNAs act as a competitive endogenous RNA sponge miRNAs and regulated transcription of mRNA. Such as circRNA-UBAP2 acts as a sponge of miR-143 to promote osteosarcoma progression [20]. Our study indicated that circRNA-UBAP2 targeted miR-382-5p and downregulated its expression level, then the expression level of PRPF8 was increased, which relevant to progression of ovarian cancer.

Alternative splicing of mRNA precursors is an important process in eukaryotes. PRPF8 is the core protein of splicing, the mutation of PRPF8 could cause the death of model cell, and it has an important relationship with the occurrence of the disease particularly in cancer [21]. Recent study reported that knockdown of PRPF8 significantly impairs mitophagosome formation, thus, these findings demonstrate that PRPF8 is essential for mitophagy and suggest that dysregulation of spliceosome-mediated mitophagy may contribute to pathogenesis of disease such as cancer [22]. PRPF8 is a highly conserved pre-mRNA splicing factor and a component of spliceosomal small nuclear ribonucleoproteins (snRNPs), which is essential in RNA and mRNA splicing through transesterification and spliceosome processes [23]. It was reported that the events of splicing were related to tumor progression, the alternative splicing of different pre-mRNAs is altered during oncogenic progression with the concomitant development of cancer features, like an increase in vascularization, cell proliferation, and invasion [24].

## Conclusions

To sum up, our study found that circRNA-UBAP2 was highly expressed in ovarian cancer tissues and cell lines. Besides, knockdown of circRNA-UBAP2 prevented the deterioration of ovarian cancer. In addition, overexpression of circRNA-UBAP2 promoted malignant behavior of ovarian cancer. Furthermore, circRNA-UBAP2 acted as an oncogene in ovarian cancer, and circRNA-UBAP2 acted as a ceRNA to sponged miR-382-5p, increased the expression level of PRPF8, and prompted proliferation and inhibited apoptosis in ovarian cancer cells. Our study might provide a new target and theoretical basis for diagnosis and therapy of ovarian cancer.

## Materials and methods

### Tissue samples

The ovarian cancer tissues and adjacent tissues were collected from 20 patients who received no radiotherapy and chemotherapy before surgery. The samples were collected for RT-qPCR assay to detected expression of circRNA-UBAP2, miR-382-5p and PRPF8. This study was approved by the First People’s Hospital of Yunnan Province. All specimens were immediately snap-frozen in liquid nitrogen after surgical remove.

### Cell culture and transfection

Normal ovarian epithelial cells (IOSE80, No. EC-H049) and ovarian cancer cells (SKOV3, No. KL; OVCAR-3, No. H-OVCAR-3; ES-2, No. H-ES-2 and A2780, No. H-A2780) were purchased from Shanghai Kanglang Biotechnology Co., Ltd.(China). Cell culture was performed using DMEM (No. PM150270, Yaji, Shanghai, China) with 10% FBS (No. 10099141, Gibco, USA) under standard culture conditions (37 °C, 5% CO_2_).

circRNA-UBAP2 cDNA cloned into vector, sh-UBAP2, miR-382-5p mimics, si-PRPF8 and their negative controls were synthesized by Ribobio (Guangzhou, China) and transfected into cultured OVCAR-3 and ES-2 cells using Lipofectamine 2000 (No. 11668–109, Invitrogen, USA) according to instructions.

### RT-qPCR

Total RNA was extracted using TRIZOL reagent (No. S308876, Yeyuan, Shanghai, China), and transcribed into cDNA using RT reaction kit (No. DD546862, Yiji, Shanghai, China). RT-qPCR was conducted using ABI system (No. 9700, USA). Using U6 as an internal control. Expression fold changes were determined using the 2^-△△CT^ method. Primers were as follows, circRNA-UBAP2 forward primer: 5′-AGCCTCAGAAGCCAACTCCTTTG-3′, and reverse primer: 5′-TCAGGTTGAGATTTGAAGTCAAGAT-3′. miR-382-5p forward primer: 5′-ATCCGTGAAGTTGTTCGTGG-3′, and reverse primer: 5′-TATGGTTGTAGAGGACTCCTTGAC-3′. PRPF8 forward primer: 5′-ATATGGCCGGAGTGTTTC-3′, and reverse primer: 5′-CACCCCCTTTGAGTGTC-3′. U6 forward primer: 5′-CTCGCTTCGGCAGCACA-3′, and reverse primer: 5′-AACGCTTCACGAATTTGCGT-3′.

### Western blotting

Each group of cells were collected and lysed in RIPA buffer (No. 2114–100, BioVision, USA) supplemented with protease inhibitor cocktail (No. S25910, Yeyuan, Shanghai, China). The concentration was determined using a BCA protein assay kit (No. orb-EHJ033662, BIOHJSW, USA). Equal amount of the extracts were loaded and subjected to SDS-PAGE (No. YB100915–12, Ybscience, Shanghai, China), transferred onto nitrocellulose membranes (No. B500, ABM GOOD, CAN), and then blotted. Antibodies specific to PRPF8 (1:1000 dilution) (No. P104217, KAB, Shanghai, China), Bcl-2 (1:1000 dilution) (No. P202729, KAB, Shanghai, China), Bax (1:1000 dilution) (No. P202573, KAB, Shanghai, China), caspase-3 (1:1000 dilution) (No. K4221, KAB, Shanghai, China), and GAPDH (1:1000 dilution) (No. P202453, KAB, Shanghai, China), and secondary antibodies (No. 20774, Millipore, USA) were reported previously.

### CCK-8

Cell proliferation was examined using CCK-8 kit (No. DD546592, Yiji, Shanghai, China) following the instructions. Briefly, each group of ovarian cancer cells were plated at a density of 1 × 10^4^ cells per well in 96-well plates with 10% FBS, and then cultured in incubators with 37 °C, 5% CO_2_ for 48 h. A total of 10 μl of CCK-8 was added to each well at 0 h, 24 h, 48 h, 72 h and 96 h, and 96-well plates were incubated for additional 1 h, then the absorbance (OD) at 450 nm wavelength was measured by enzyme labeling instrument (No. DXT-11008412001, Roche, USA).

### Flow cytometry

Cell apoptosis rate and cell cycle distribution of each group of ovarian cancer cells were measured using flow cytometry. Fix cells in cold citrate buffer (No. orb-EHJ024527, BIOHJSW, USA) for 10 min at 4 °C to make the cell density 2 × 10^5^ cells/200 ml citrate buffer then Transfer cells to a Falcon tube. PE Annexin V apoptosis detection kit (No. 640934–1, Biolegend, USA) was used by determining the relative amount of AnnexinV-FITC-positive, PI-negative cells. Cells were stained with PI using the PI cell cycle kit (No. CSK-0112, Nexcelom Bioscience, USA). Deal with the cells according to instructions and then run FAScan detects cell apoptosis rate and cell cycle distribution.

### Dual-luciferase reporter gene system

The circRNA-UBAP2 and PRPF8 3’UTR binging sites of miR-382-5p were predicted by starBase. The reporter plasmids were obtained by inserting circRNA-UBAP2 and PRPF8 3’UTR binging sites into the pmirGLO vector (No. YB-0577, Ybscience, Shanghai, China). miR-382-5p mimics and reporter plasmids were co-transfected into 293 T cells via Lipofectamine 2000. Firefly and Renilla luciferase activities were measured after cultured for 48 h using the dual-luciferase Reporter Assay System (No. E1910, Promega, USA) according to instructions.

### Statistical analyses

All experiments in this study were repeated three times. The results were expressed as mean ± SD, and statistical analysis were completed by *t*-test or one-way ANOVA using SPSS 17.0. *P*<0.05 was considered statistically significant.

## Data Availability

All data generated or analysed during this study are included in this published article.
